# Occlusion Handling for Mobile AR Applications in Indoor and Outdoor Scenarios

**DOI:** 10.3390/s23094245

**Published:** 2023-04-24

**Authors:** Muhammad Alfakhori, Juan Sebastián Sardi Barzallo, Volker Coors

**Affiliations:** Centre for Geodesy and Geoinformatics, Stuttgart University of Applied Sciences (HFT Stuttgart), 70174 Stuttgart, Germany

**Keywords:** augmented reality, mixed reality, occlusion, urban planning, AEC, HoloLens, 3D city model, CityGML

## Abstract

When producing an engaging augmented reality (AR) user experience, it is crucial to create AR content that mimics real-life objects’ behavior to the greatest extent possible. A critical aspect to achieve this is ensuring that the digital objects conform to line-of-sight rules and are either partially or completely occluded, just like real-world objects would be. The study explores the concept of utilizing a pre-existing 3D representation of the physical environment as an occlusion mask that governs the rendering of each pixel. Specifically, the research aligns a Level of Detail (LOD) 1 building model and a 3D mesh model with their real-world counterparts and evaluates the effectiveness of occlusion between the two models in an outdoor setting. Despite the mesh model containing more detailed information, the overall results do not show improvement. In an indoor scenario, the researchers leverage the scanning capability of HoloLens 2.0 to create a pre-scanned representation, which helps overcome the limited range and delay of the mesh reconstruction.

## 1. Introduction

Augmented reality (AR) is a technology that blends digital information, such as images, sounds, and other sensory inputs, with the real world in real time. It involves using a camera and a display device, which could be a smartphone, tablet, or AR headset, to overlay virtual objects and information onto the physical environment. In other words, AR allows users to view contents and objects that they would otherwise be unable to see [1].

AR can be extended further to provide a more interactive and immersive experience by introducing interaction between digital content and the real-world environment, achieving a mixed reality (MR) and near-real experience. This necessitates a seamless transition between virtual and physical environments, as well as the ability for both to coexist and interact realistically. However, simulating physical interactions, such as collisions, shadows, lighting, and occlusions, is a difficult challenge to overcome [1,2]. Therefore, digital content should align with the real environment, including position, scale, and occlusion by other objects.

**Figure 1:**
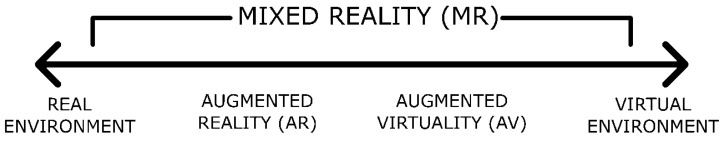
Reality–virtuality continuum (adapted from [3]).

Milgram et al. [3] presented the reality–virtuality continuum, a theoretical model (shown in Figure 1) that describes the different levels of immersion and interaction between the physical world and virtual environments. It spans from the real world (reality) on one end to fully immersive virtual environments (virtuality) on the other. The continuum includes various levels of augmented reality, mixed reality, and augmented virtuality depending on the extent of digital content presence.

The reality–virtuality continuum shows the trade-offs between immersion and real-world interaction, which helps designers create immersive experiences. Immersion and interaction may vary by use case and experience.

**Figure 2:**
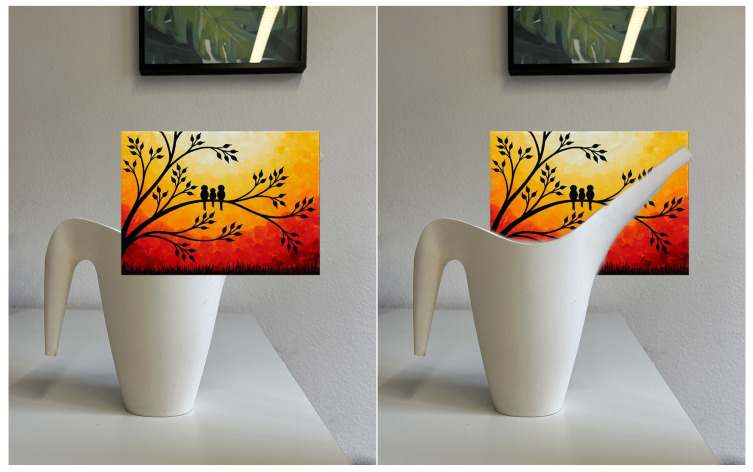
Demonstration of the occlusion effect. (**left**) Occlusion disabled. Occlusion enabled (**right**).

The objective of occlusion in AR scenarios is to ensure compliance with the laws of line of sight. This means that virtual objects positioned behind real-life objects should be concealed or hidden from view to provide a more authentic experience for viewers and enhance their perception of depth, as shown in Figure 2. Neglecting to apply the occlusion effect results in the digital content appearing to float above the scene rather than being part of it, as demonstrated in Figure 2. However, when the occlusion effect is employed, portions of the physical objects are obscured, resulting in a more natural-looking scene, where the digital content merges seamlessly with the real-world environment, creating a more realistic experience.

To achieve occlusion, a 3D model of the physical environment is required as a basis for concealing virtual objects. This representation functions as a mask to hide the digital content. To create this real-world model, real-time 3D sensing methods can be used, or a pre-existing city model in a generalized format can be used. Both methods achieve the same outcome.

AR has significant applications in both indoor and outdoor settings. In indoor AR, digital content is superimposed onto the physical space of a closed environment, such as a room or building. In contrast, outdoor AR is used to enhance experiences in open areas, such as parks or streets. The difference between the two lies in the environmental factors that influence the user’s experience, such as lighting and space limitations. While indoor AR can provide a more controlled and structured environment for users, outdoor AR often requires more advanced tracking and localization technologies to accurately place digital content in the user’s view. Overall, both indoor and outdoor AR have vast potential in numerous fields, including education, entertainment, and navigation, offering innovative and engaging ways to interact with the world around us.

Given the crucial role urban planning plays in our daily lives, emerging technologies such as AR have the potential to make significant contributions to the field. To expand beyond the current limited range of two-dimensional data visualization or marker-based systems, it is imperative to increase the use of qualitative augmented reality solutions [4].

### Objective

Building on their prior research [5], this research aims to develop an augmented reality participation tool that can handle occlusion effectively in both indoor and outdoor environments. This was accomplished by studying the occlusion problem and utilizing various data sources to function as an occlusion mask. The study compares the effectiveness of the mesh model and LOD1 building model for occlusion handling in terms of quality and processing requirements and provides recommendations on their optimal use in different scenarios. Furthermore, this research aims to address the discrepancies between indoor and outdoor AR occlusion handling to improve the overall usability and effectiveness of the tool.

## 2. State of the Art

### 2.1. Outdoor AR

To visualize digital content accurately based on the user’s intended location in an outdoor environment, it is essential to determine the user’s position and viewpoint. Tracking technologies can be classified into three types: sensor based, computer vision based, and hybrid. Sensor-based tracking involves using GNSS sensors, magnetometers, and gyroscopes to determine the camera position and field of view (FOV) [1,6]. Although this solution’s mobility makes it ideal for outdoor AR, research indicates that GNSS accuracy limits the user’s experience and may result in an incorrect visualization [1,4,7]. To improve accuracy, a wide-area, high-precision RTK GNSS tracking system can be implemented [8].

The computer-vision-based tracking, on the other hand, uses camera frames to estimate where the object is and how it is moving [9]. The drawback to this approach is that the markers have to be visible at all times, and other elements of the environment must not block their view. Ref. [10] introduceda direct integration of large 3D point clouds with semantics in a web-based, markerless mobile AR application for real-time visualization. A recent study [11] proposed an end-to-end network, Siam2D3D-Net, to jointly learn local feature representations for 2D image patches and 3D LiDAR point cloud volumes by using mobile laser scanning (MLS) data from the urban scene and provides a precise 2D–3D patch–volume dataset that contains paired matching 2D image patches and 3D LiDAR point cloud volumes. The results show that the proposed Siam2D3D-Net can match and establish 2D–3D correspondences between the query 2D image and the 3D LiDAR point cloud reference map.

The hybrid-based tracking approach is a combination of the two tracking methods that came before it. In this approach, the readings from the sensors are used to initiate the tracking process, and further improvement can be achieved through the use of computed vision [12]. A hybrid combination that includes inertial measurement unit (IMU) sensors and a GNSS receiver with computer vision in order to improve GNSS accuracy is one of the methods that can be used in this context [13].

Ref. [12] discussed AR in-vehicle infotainment (AR-IVI), which involves the integration of digital information to assist drivers in navigating and learning about their surroundings, and may also be extended to entertain passengers. The study demonstrates the system structure and highlights the need to integrate different sensors, as visual sensing systems may not function properly in hazardous weather conditions. In such situations, the system must be able to detect and reject low-quality data to ensure safety.

A new approach for large-scale outdoor AR involves using a 3D city model that includes terrain and building models. These models are processed into meshes to align the scene with the real world, which requires an initial alignment from the user. With this method, mobile devices can be accurately registered worldwide, enabling effective and precise AR experiences [14].

Ref. [15] presented a hybrid and lightweight solution for the 3D tracking of arbitrary geometry for use in AR scenarios that take place outside. In order to validate and improve tracking performance in large-scale and uncontrolled outdoor environments, the camera pose information obtained by the ARCore Software Development Kit (SDK) and the vSLAM algorithm is combined with the semantic and geometric output of a convolutional neural network (CNN) object detector. The methodology consists of the following three primary steps: (i) the real-time detection, segmentation, and localization of the region of interest (ROI) in camera frames; (ii) the computation of 2D–3D correspondences to enhance pose estimation of a 3D overlay; and (iii) the training of the Mask R-CNN model to extract the class, bounding box, and mask predictions.

### 2.2. Occlusion Handling in AR

Occlusion is a highly effective 3D cue; in fact, it is the most fundamental depth indicator [16]. Ref. [17] provided a comprehensive review of 161 articles on AR occlusion handling published between January 1992 and August 2020. The occlusion problem can be summarized into the order problem, the X-vision problem, and the visual display problem. The first and most important problem to be solved is the order problem, in which the rendering order of various objects is determined to establish whether each pixel’s value is derived from the real world or the augmented image.

Ref. [2] presented a model-based approach, where actual items are wrapped in an approximate bounding box and the tracking camera is used to locate the real target. Then, a segmentation mask is generated by subtracting the background. On the other hand, Ref. [1] used the Open Street Map (OSM) building footprint and extruded it with generic values to create a mask model for outdoor occlusion. However, the generalization of the building’s height provided an inaccurate occlusion effect.

By contrast, Ref. [5] utilized the Level of Detail 1 (LOD1) CityGML building model to generate an occlusion mask. The occlusion model is aligned with the real-world equivalent by means of a computer vision approach based on feature point detection, which helps to maintain the position of the mask model.

In their work, Ref. [18] proposed an airborne AR solution that uses drones to visualize different landscape scenarios, while making use of the existing twin model as an occlusion mask. The method involves using a detailed mesh city model, which enables them to achieve a precision of approximately 0.80 using intersection over union. Due to the complexity of the occlusion model, the video stream is processed first at the server and then delivered to the user with a delay of 3 s.

The depth-based technique involves using live data to capture and estimate the depth of the real world. There are various methods for acquiring the live depth model, including using a stereo camera to reconstruct depth [2]. Monocular images can also be used, as shown by [19], who utilized monocular SLAM to reconstruct the assembly scene. The rebuilt sparse 3D points are then transformed into depth points in the depth map. Although the proposed method achieved accurate results, it required a dedicated GPU to perform real-time SLAM, which limited the solution to GPU-enabled devices.

Another approach to depth-based occlusion handling is demonstrated by [20], who used AI to perform instance segmentation to extract individual buildings and then compared them with map data to determine the distance between the user and each building. However, this method shows inaccuracy during user movement, as the FOV is updated and new segmentation is required. Ref. [21] took a different approach and registered RGB-D images to the real world using FAST-ICP-ORB to determine which part of the virtual object to render. This approach requires extra hardware, such as a Microsoft Kinect, to generate the RGB-D images. In comparison to the previous methods, the hardware requirement may limit the application of this approach.

### 2.3. AR for Urban Planning and Architecture

Urban planning experts can navigate city streets while projecting 3D virtual structures through a wide range of AR applications to increase the level of immersion in urban planning solutions. According to [22], interactive solutions can enhance public engagement in urban planning procedures significantly. To achieve this, a smartphone application was developed that overlays the proposed building design on the real-world environment, allowing viewers to inspect and provide feedback on the suggested 3D architectural model. As a result, the graphical user interface (GUI) was designed to be as simple and intuitive as possible, with smartphone familiarity having a direct impact on the user experience.

The City 3D-AR pilot project addresses the challenge of placing 3D objects in real-world environments by utilizing GPS longitude and latitude data. However, accurately representing large 3D objects in external environments and recognizing building locations based on viewer distance and angle pose significant challenges. The conventional fiducial marker technique, typically used for indoor applications over short distances, is not suitable for outdoor use [4]. In this project, a processing and rendering laptop connected to a USB GPS sensor was used to achieve accurate positioning. The Vuzix Wrap 920AR AR head-mounted display (HMD) was utilized to visualize the output. The user’s location is continuously tracked to enable viewer movement and automatic 3D object transformations, rotations, and scaling in response to changes in viewing angle and distance. To provide an interactive AR experience, a database of 3D buildings was included, enabling users to modify architectural models.

Following the 2011 earthquakes in Christchurch, CityViewAR was developed to display panoramic images of the city [6]. The application was designed to aid in the reconstruction of destroyed structures and plan for future urban growth. To navigate the panoramas, a GPS-enabled smartphone is required. The panoramic visuals rotate as the viewer rotates the device’s gyroscope sensor, providing an immersive and interactive experience.

Communicating design ideas between architects and end users in the context of architectural design has always been a challenging and time-consuming task. Various tools and techniques, such as sketches and scale models, have been utilized throughout history to address this challenge [23,24]. However, until the advent of current technologies, such as CAD and BIM, they were limited to two-dimensional representations, which reduced the intuitiveness of the process [25]. The integration of extended reality (XR) technologies with BIM has emerged as a promising tool for optimizing the communication of design ideas among professionals and stakeholders throughout the architecture, engineering, and construction (AEC) industry process [26].

## 3. Methodology

Most of the research on occlusion handling aims to achieve precise results at the pixel level. To achieve this objective, researchers use either real-time 3D sensing technology at a small scale or a pre-existing model of the environment in their studies. However, both of these methodologies have not been able to integrate effectively into their findings.

Moreover, prior research requires additional hardware or external processing to create the occlusion mask, which limits the mobility of the application. As a result, developing a method that can accurately handle occlusion in real time without additional hardware or external processing would be a significant step forward in this field.

The current study proposes a technique to enhance augmented reality applications by integrating real-world 3D models as an occlusion mask. The proposed method aims to overcome the limitations of real-time scanning, such as the range and transparency of materials, such as window glass, and provide a more accurate representation of the augmented environment. By aligning the 3D model with the real environment in real time, the approach aims to minimize the discrepancies between the virtual and real-world objects, enhancing the overall immersion and realism of the augmented environment. The method has the potential to find applications in various fields, such as urban planning, architecture, and interior design, improving the user experience and the efficiency of the design process.

### 3.1. Platform

The AR occlusion handler was developed using the Unity game engine and the Mixed Reality Toolkit (MRTK) AR SDK, while the Microsoft HoloLens 2.0 platform was used for 3D real-time sensing and AR application development. The onboard ToF camera, which has a limited range of 5 m, was used for this purpose.

### 3.2. Data Preparation

The occlusion mask for the AR application was created by utilizing the CityGML building model LOD1 and the 3D mesh model provided by the Surveying Measurements Office of the City of Stuttgart (Stadtmessungsamt Stuttgart). Since CityGML data are stored in a text-based format, they had to be converted to a 3D mesh OBJ format to be loaded into the game engine. To achieve this, an attribute filter based on the block number was used to extract the buildings, followed by an OBJ writer to provide a mesh output using the FME Workbench.

Even though both CityGML and 3D mesh models use real-world coordinate systems based on UTM projection (UTM/ETRS89 EPSG:25832) with the *Z*-axis pointing upward, a transformation is necessary to generate a local coordinate system that is suitable for computer graphics. When using the real-world coordinate system in computer graphics, a problem arises because the *Z*-axis extends toward the user and beyond the screen, leading to the presentation of the wrong side of the building.

In this instance, the transformation matrix presented below involves a rotation around the *X*-axis and a translation to the origin point:

$$\left[\begin{array}{cccc} 1 & 0 & 0 & -513000.0\\ 0 & 0 & 1 & -5402000.0\\ 0 & -1.0 & 0 & -256.0\\ 0 & 0 & 0 & 1 \end{array}\right]$$

**Figure 3:**
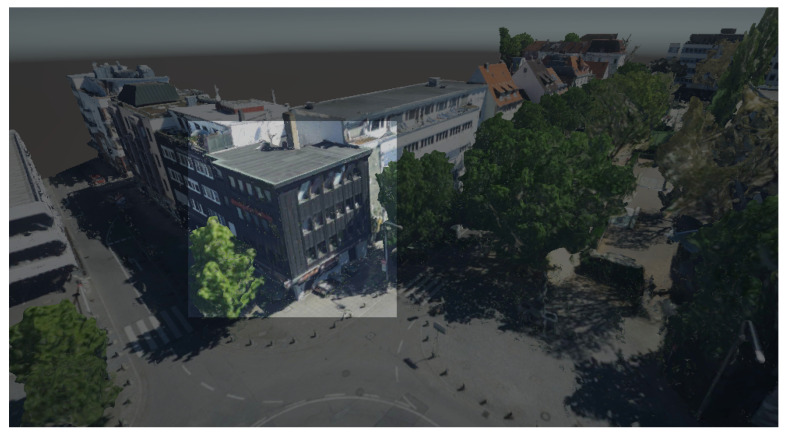
Extracted building from the 3D mesh model.

The 3D mesh model that covers the entire study area contains information beyond the scope of building geometry, such as trees and streets, which are not necessary for occlusion purposes. Therefore, manual mesh segmentation is required to extract the buildings. As shown in Figure 3, the extracted buildings are highlighted in the mesh of the study area.

### 3.3. Study Cases

The significant horizontal and vertical distances pose a limitation, as objects that fall outside the scanning range of the device will not be considered in the occlusion mask based solely on the scanned data.

**Figure 4:**
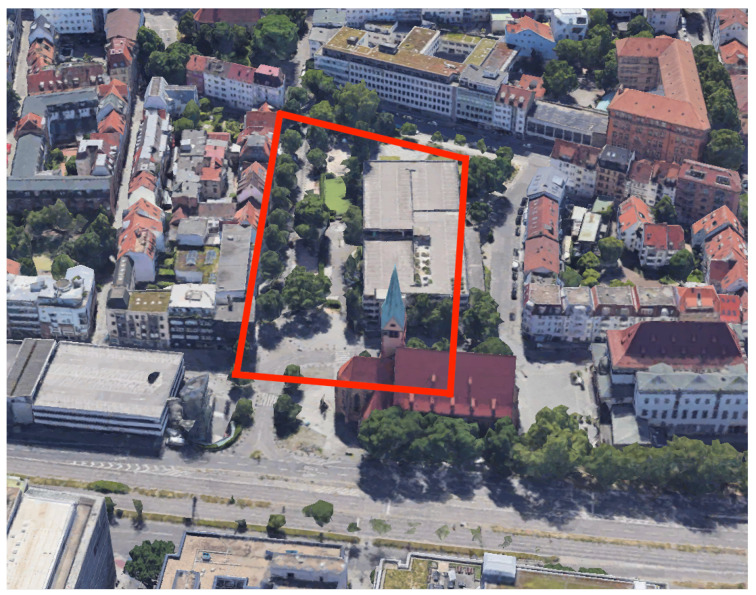
Bird’s eye view of the Leonhardsviertel study area. Image source: Google Earth.

The Leonhardsviertel area in the city of Stuttgart, depicted in Figure 4, was chosen as an outdoor case study as part of iCity2: Streetmoves4iCity (https://www.hft-stuttgart.de/forschung/projekte/aktuell/icity2-streetmoves4icity, accesed on 16 March 2023). The project’s objective is to encourage sustainable mobility and improve the urban space by implementing a car-free environment. To achieve this, occlusion masks were created based on LOD1 and 3D mesh models, which were then aligned with the actual buildings in the study area. The process of creating each mask is compared, and the results of both masks are evaluated.

Despite the indoor environment being more regulated, it presents its own challenges, including range limitations that persist indoors. The presence of various material types in the scene can impact the scan, and transparent windows can cause inaccuracies in the representation of the scan.

**Figure 5:**
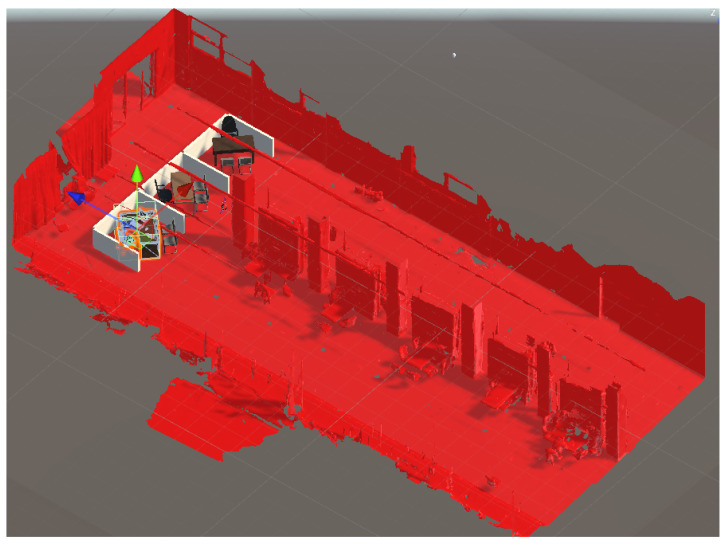
Office design aligned with the 3D scanned mesh used as occlusion mask.

The indoor study was conducted in a space that covers approximately 309 square meters, with dimensions of roughly 27.76 m by 11.12 m. The objective of the study was to simulate a real-world office layout design scenario with enough space to render three office modules at a 1:1 scale. In the users’ study mentioned in Section 5.3, users were given the opportunity to actively participate in the design rectification process by manipulating digital furniture and structural components. To obtain the 3D mesh of the room, the Microsoft HoloLens 2 Spatial Mapping feature was utilized, given the size of the room. Imperfections captured during the scanning process were subsequently rectified through mesh editing, and the mesh was exported for integration between the design draft made on BIM and the real-world scanned mesh, as seen in Figure 5, which served as a reference for the design draft. Finally, the occlusion material was attached to the 3D mesh to create an occlusion mask for the scene.

## 4. Implementation

### 4.1. Occlusion Shader

The occlusion shader is responsible for determining the appearance of each pixel on the display. This shader is contained within a material that can be applied to either the 3D building model or the 3D mesh model as an occlusion mask. To ensure that the occlusion mask is computed before any other meshes in the scene, the Queue value should be set to one unit below the geometry value as shown in Listing 1. The geometry value, which is always set to 2000, specifies the priority for displaying solid objects. The occlusion mask pixels are written to the depth buffer when ZWrite is enabled, while the ZTest is configured to render only the geometry that is in front of other objects, and not any pixels behind them. This means that the occlusion mask prevents all objects located behind it from being rendered. Additionally, the ColorMask is set to zero, which allows the shader to adjust all three RGB values as well as the alpha channel that represents transparency. The HoloLens adds colors together, so black appears translucent. When the geometry of the building model, which acts as an occlusion mask, is drawn in the scene, any object that is hidden behind it will be obscured.

**Listing 1.** Defining the rendering queue.
1   Shader 
"Occlusion"
2   {
3       SubShader
4       {5           Tags { "Queue"="Geometry-1" }
6           ZWrite On

7           ZTest LEqual

8           ColorMask 0


Lines 15–31 in Listing 2 define the necessary structure for storing position data input and output. The POSITION parameter is utilized for transmitting the 32-bit 3D coordinates of each pixel, whereas SV POSITION maintains the screen coordinates of the object and the Z value provided by the ZTest.

**Listing 2.** Necessary structure for storing position data input and output.
15          CGPROGRAM

16          #pragma vertex vert

17          #pragma fragment frag

18

19          #include 
"UnityCG.cginc"

20

21          struct appdata

22          {

23              float4 vertex : POSITION;

24              UNITY_VERTEX_INPUT_INSTANCE_ID

25          };

26

27          struct v2f

28          {

29              float4 position : SV_POSITION;

30              UNITY_VERTEX_OUTPUT_STEREO

31          };


Lines 33–38 Listing 3 contain the fundamental operation to convert vertex object-space 3D coordinates into 2D screen coordinates. Additionally, lines 40 to 42 implement a fixed4 method that produces a black color value for each vertex during the screen rendering process.  

**Listing 3.** Convert vertex object-space 3D coordinates into 2D screen coordinates.
33         v2f vert (appdata input)

34             {v2f output;

35             UNITY_SETUP_INSTANCE_ID(input);

36             UNITY_INITIALIZE_VERTEX_OUTPUT_STEREO(output);

37             output.position=UnityObjectToClipPos(input.vertex);

38             return output;}

39

40         fixed4 frag (v2f input) : SV_Target

41         {return fixed4(0.0, 0.0, 0.0, 0.0);}

42         ENDCG

43     }

44 }


### 4.2. Alignment of the Occlusion Mask

To align the occlusion mask with the physical environment, the spatial anchor from the MRTK SDK is employed to establish a reference point and its relation to other detected keypoints. This involves utilizing a set of anchors that are automatically generated and distributed. Once the entire 3D scene is aligned with the real environment based on these anchors, a camera component transformation correction is performed.

The “Spongy State” was the initial state of the application, where the current head tracking data and spatial anchor served as a dynamic input for the world looking tool. In this state, the origin point of the local coordinate system was based on the head pose. Then, an optimization engine was used to align the 3D scene with the real world by applying rotation and translation, while maintaining a consistent scale. The resulting transformation was saved in a “Frozen State” to ensure persistence across different application sessions.

## 5. Results and Evaluation

### 5.1. Outdoor Case Study

**Figure 6:**
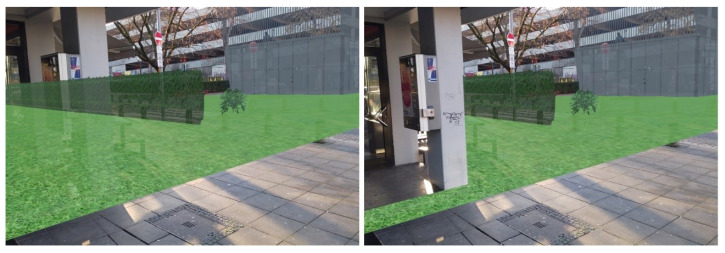
Occlusion based on the 3D mesh building model. (**left**) Disabled. (**right**) Enabled.

**Figure 7:**
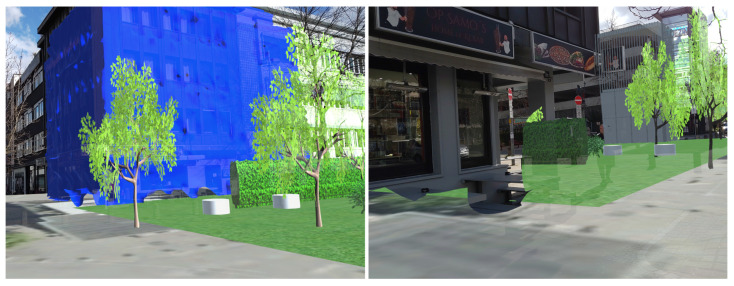
(**left**) Alignment of the 3D mesh model with actual building. (**right**) Occlusion based on the 3D mesh model.

For the outdoor case study, both the LOD1 and 3D mesh building models were appropriately aligned with the actual buildings in the study area. Figure 6 presents the occlusion effect based on the3D building model that was used as an occlusion mask. On the left side, the 3D design can be seen floating over the genuine building when occlusion is disabled. On the right side, the scene depicts how it appears when the occlusion handling is activated. The occlusion mask is precisely aligned with the real-life counterpart, causing augmented content to be obscured along the building’s edge. The occlusion mask’s edges appear crisp since the building’s geometry was represented using straight edges. On the other hand, Figure 7 shows the occlusion effect is based on the 3D mesh model where the edges of the buildings are not presented with straight lines.

### 5.2. Indoor Case Study

The digital elements had to be organized considering the pre-existing furniture and the central walls of the room; nevertheless, the time delay and the changing depth of the spatial mesh reconstruction caused continuous failures of occlusion and collision of digital objects with real-world ones. As a result, it produced an end-user interaction that was less fluid and intuitive.

**Figure 8:**
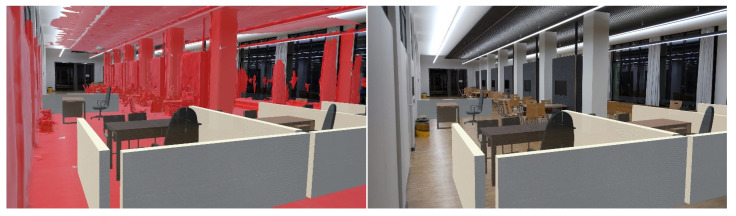
Alignment of pre-scanned mesh with real world.

**Figure 9:**
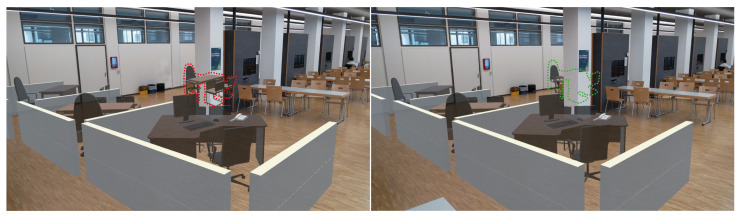
Occlusion effect based on the pre-scanned mesh. (**left**) Disabled. (**right**) Enabled.

Figure 8 shows the alignment of the pre-scanned mesh with the indoor scene and the occlusion effect is presented in Figure 9.

### 5.3. Users’ Study

Since the occlusion effect is primarily a visual phenomenon, evaluating it relies on conducting a user study, which is a crucial component of the user-centered design process.

During the user study, two user groups participated, with the first group consisting of 18 individuals from diverse backgrounds and with varying levels of experience in AR, in the outdoor experiment. The second group of 20 individuals comprised an equal number of males and females (10 each) and ranged in age from 18 to 57 years old. In total, 40% of the participants were AEC-related students, 25% were students from other fields, such as computational design and industrial engineering, 25% were AEC-related professionals, and 10% were professionals from other fields. Although the age range of the participants was fairly evenly distributed, it was interesting to note that 60% of the participants had previous similar experiences related to the use of augmented reality. In part, this reflects the rapid adoption of augmented reality technologies today.

The two experiments began with a brief introduction to the technology and the research’s objective, followed by participants being given the opportunity to explore the scene independently. Subsequently, feedback from users was collected using a questionnaire, and individual comments were also recorded.

In total, 86% of the first group and 68.4% of the second group reported having a “positive overall impression” in their feedback. In contrast, only 7% of the first group disagreed. When asked about the immersion of the experience, 86% of the first group agreed, whereas 14% had a neutral or unfavorable impression. Comparable responses were collected from the second group on the immersion.

During the outdoor experiment, some users reported that the visibility of the augmented content was unclear and they were unable to explore the entire scene, depending on the ambient light. Additionally, a few users commented on the quality of the content, describing it as not appearing realistic.

In order to evaluate the overall aim of the AR tools, which is to enable users to participate and take an active role in the design process, the second group was asked if they thought that using this technology would make it easier for a client to approve a design draft faster than with traditional methods. This was done to find out how professionals feel about new technologies compared to traditional methods. In general, the answer was positive, with 66.7% strongly agreeing with the idea, 27.8% agreeing, and 5.6% being neutral.

## 6. Discussion

This study’s results indicate that various real-world 3D representations can serve as effective occlusion masks, thereby enhancing the immersive quality of the AR experience. However, the quality of the occlusion effect may vary depending on the data sources used and may necessitate different pre-processing techniques. It is worth noting that this research underscores the importance of selecting appropriate data sources and employing proper pre-processing techniques to ensure high-quality occlusion effects. Such measures can greatly improve the overall user experience and increase the effectiveness of AR tools in urban planning and the AEC industry. These findings can inform the development of more advanced AR technologies that incorporate high-quality occlusion effects and provide users with more realistic and engaging experiences.

The study’s findings indicate that the use of spatial anchors in conjunction with world locking tools is an effective way to anchor digital content to the real-world coordinate system in the study area. Furthermore, the study confirms the reliability of the computer vision-based tracking system, which ensures stable tracking of the user’s position throughout the application’s usage. These results are consistent with previous research, such as that conducted by [27]. The alignment process proposed in this method is consistent with the approach outlined in [14], which involves both initial and fine alignment to achieve precise localization.

Through a comparison of the occlusion visual effect between the LOD1 building model and 3D mesh, it becomes evident that the former offers cleaner occlusion, as the geometry of buildings is represented by straight edges and fewer noises appear in the mesh itself. In contrast, the mesh model comprises a vast number of discrete polygons. Although the mesh model provides more data beyond the buildings, it necessitates segmentation to extract various features, as the mesh does not have a semantic representation.

In spite of that, the mesh model offers a representation of roof shapes; this information is often unnecessary, as the user typically views the environment from a ground perspective. In contrast, the LOD2 building model can provide comparable output. Additionally, the high cost of capturing and generating the mesh model, along with its limited availability, may restrict the application’s transferability to other study areas.

Unlike the approach in [1], utilizing the aforementioned data does not necessitate any generalization, which could result in an imprecise portrayal of building heights. Compared to the method proposed in [18], our method runs in real time and does not require external processing, which can cause delays in displaying the effect.

There are some factors that may affect the effectiveness and usability of occlusion handling tools in indoor and outdoor cases. For instance, the lighting conditions and environmental factors may vary significantly in outdoor environments, making it more challenging to accurately detect and handle occlusion. Additionally, the tracking and localization technologies required for outdoor AR may be more complex, which could potentially impact the performance and usability of occlusion handling tools. However, the usability of these tools is also dependent on the specific application and use case.

Although ToF technology is capable of scanning up to five meters, its range may not always be sufficient. In the case of our study, where users interact with digital objects and move them around, the ToF scanner’s limited range may result in incomplete scans. This issue becomes more prominent when the application is utilized in large indoor areas, where there is a higher probability of the scanner being unable to capture the entirety of the environment. Incomplete scans can lead to inaccuracies in the representation of the environment, hindering the application’s ability to provide an immersive and realistic user experience. Thus, having a pre-scanned version of the environment can help alleviate this issue.

In contrast to prior studies [18,19,20], a notable strength of this research is that it does not necessitate any additional hardware or software. The occlusion mask and actual pixel rendering are both calculated using the device itself, while even the pre-scanned mesh can be obtained using the same hardware (HoloLens).

To ensure a high-quality user experience, it is crucial to prioritize the content quality and realism of the three-dimensional design employed. This can be accomplished by incorporating pre-existing elements from the study program, such as selecting tree and grass types that complement the existing environment. Another critical consideration is the brightness of the glasses. Negative feedback was received during sunny weather, as it negatively impacted the overall impression and clarity of the experience. Hence, it is necessary to use designs that are as clear as possible to enhance the user experience.

Overall, participants responded positively to the topics presented in the study. However, there were some difficulties in using the application. While the HMD provides an immersive environment, where users can move through scenes and interact with digital objects naturally, object translation and rotation may not be as intuitive as expected. Although beginners may require some practice before object movement becomes more natural, it is not a particularly difficult task.

Despite the difficulties in handling the application, the results indicated that participants enjoyed the experience, which is not typical for an activity that requires a considerable degree of difficulty for some users. Moreover, the application elicited playful behavior among the users, encouraging a game-like experience that captured their attention and invited them to intervene and make changes more smoothly.

## 7. Conclusions

The research proposes the idea of improving the way occlusion is managed by making use of different 3D representations, such as the LOD1, 3D mesh model, and pre-scanned model, to serve as an occlusion mask. By aligning the geometry of the building model with its real-life equivalent, the occlusion shader determines the rendering of each pixel. The selection of 3D data may vary depending on the specific situation. While LOD1 is simpler to employ and yields more favorable outcomes, the mesh model can incorporate additional details about the roof and other building characteristics. An application for urban planning and architectural design has been developed using the suggested technique, which enables users to engage in the decision-making process and integrate feedback from the local community into future scenarios. To evaluate the effectiveness of the occlusion screening effect proposed in the study, a test was conducted, involving users from various backgrounds who assessed the application and evaluated different occlusion options. The majority of participants observed that activating the occlusion option improved their perception of depth and created a more immersive experience.

Subsequent research efforts will concentrate on automating the creation of the occlusion mask through the availability of diverse data and user pre-selection. To enhance user engagement, the integration of virtual 3D sounds could be considered by researchers. The quick advancement of AR technologies and SDKs implies potential opportunities to develop more advanced and lifelike applications in the future. Keeping pace with these technological advancements will be a persistent challenge for researchers in this domain.

## Data Availability

The data presented in this study are available on request from the corresponding author. The data are not publicly available due to the restricted use of the CityGML building model and 3D mesh model.

## Abbreviations

The following abbreviations are used in this manuscript:

AR	Augmented Reality
AEC	Architecture, Engineering and Construction
CNN	Convolutional Neural Network
FOV	Field of View
GUI	Graphical User Interface
HMD	Head-Mounted Display
IMU	Inertial Measurement Unit
IVI	In-Vehicle Infotainment
LOD	Level of Detail
MLS	Mobile Laser Scanner
MR	Mixed Reality
MRTK	Mixed Reality Toolkit
OSM	Open Street Map
ROI	Region of Interest
SDK	Software Development Kit
SLAM	Simultaneous Localization And Mapping
ToF	Time of Flight
XR	Extended Reality
